# Green Synthesis of Highly Luminescent Carbon Quantum Dots from Asafoetida and Their Antibacterial Properties

**DOI:** 10.3390/nano15231804

**Published:** 2025-11-29

**Authors:** Zahra Ramezani, Armita Khayat, Brian De La Franier, Abdolghani Ameri, Michael Thompson

**Affiliations:** 1 Nanotechnology Research Center, Medical Basic Sciences Research Institute, Ahvaz Jundishapur Univesity of Medical Sciences, Ahvaz 61357-15794, Iran; 2 Department of Chemistry, University of Toronto, 80 St George S., Toronto, ON M5S 3H6, Canada; brian.delafranier@mail.utoronto.ca; 3 Department of Food and Drug Control, Ahvaz Jundishapur University of Medical Sciences, Ahvaz 61357-15794, Iran

**Keywords:** Asafoetida, antibacterial activity, carbon quantum dots, copper doped carbon quantum dots: surface coating, *E. coli*, *S. aureus*

## Abstract

Highly luminescent carbon quantum dots (CQDs) and copper-doped CQDs (Cu-CQDs) were synthesized from Asafoetida powder using a one-pot hydrothermal method. The structural, morphological, and optical properties of the synthesized CQDs were characterized via microscopic and spectroscopic techniques. Photoluminescence studies revealed that CQDs exhibited maximum emission at 450 nm under 335 nm excitation with a quantum yield of 37%, while Cu-CQDs showed a red-shifted emission at 455 nm under 330 nm excitation and a significantly enhanced quantum yield of 73.4%. As a proof of concept for potential biomedical and surface-coating applications, the antimicrobial activity of both CQDs was evaluated against *Escherichia coli* (*E. coli*) and *Staphylococcus aureus* (*S. aureus*). Cu-CQDs exhibited superior antibacterial efficacy, with a minimum inhibitory concentration of 0.3 mg mL^−1^. Furthermore, Cu-CQDs were immobilized on polyvinyl chloride (PVC) surface, and fluorescence microscopy confirmed their antibacterial effectiveness, demonstrating their potential for functionalized antimicrobial coatings.

## 1. Introduction

Antibiotic-resistant bacteria has triggered a global healthcare crisis, driving the need for new alternative therapeutic and preventative strategies [1]. Among various alternatives, nanomaterials with inherent antibacterial properties, such as Carbon Quantum Dots (CQDs), have shown significant promise [2]. These carbon-based nanoparticles, typically less than 10 nanometers in size, are recognized for their biocompatibility, tunable optical properties, and versatility in surface modification. CQDs have garnered attention not only for applications in bioimaging and drug delivery but also for their potential as antibacterial agents, particularly when activated by visible or natural light [3,4,5]. CQDs have emerged as a promising nanomaterial in the fight against bacterial infections, demonstrating multiple antibacterial mechanisms that enhance their efficacy [6]. CQDs target bacteria through various pathways, such as the generation of reactive oxygen species (ROS), disruption of bacterial membranes, and interference with intracellular processes. The production of ROS leads to oxidative stress, damaging bacterial proteins, lipids, and DNA. Additionally, CQDs can directly interact with the bacterial cell membrane, causing structural destabilization and leakage of essential cellular components. Their ability to penetrate bacterial cells further allows them to disrupt metabolic pathways and inhibit growth [3,6,7].

The synthesis of CQDs involves diverse methods and carbon precursors, including natural products and waste materials, which influence their functional groups and antibacterial properties [2,8,9]. The choice of carbon source plays a pivotal role in determining the elemental composition, optical properties, and bioactivity of the CQDs. For example, carbon sources rich in heteroatoms like nitrogen or sulfur enhance fluorescence and quantum yield, while those containing bioactive compounds, such as polyphenols, impart inherent antibacterial characteristics. These features make CQDs highly adaptable for biomedical applications [10,11]. Different synthetic approaches involving factors such as temperature, pressure, and reaction environment, can directly affect the size, shape, and mono-dispersity of CQDs, while also enabling specific surface modifications. Smaller, more uniform particles with consistent morphology are ideal for optical applications and biological interactions. CQDs surface groups are critical in antimicrobial applications, where compatibility and binding capability are essential. Moreover, synthetic methods vary in efficiency and yield. Techniques such as hydrothermal and microwave synthesis typically produce higher yields of functionalized CQDs with fewer impurities. In summary, while the carbon source primarily dictates the elemental composition and initial functional groups of CQDs, the synthetic method controls particle size, morphology, and surface chemistry—factors essential for tailoring CQDs to applications in imaging, drug delivery, and antibacterial treatment [4,5,12,13].

CQDs synthesized from plants, as a carbon source, in aqueous media represents a promising advancement in green chemistry and nanotechnology. For enhanced antibacterial activity, selecting plants with inherent antimicrobial properties offers additional benefits, as these plants can transfer their antibacterial agents to the surface of as-prepared CQDs. Ferula asafoetida Linn. is the primary source of asafoetida, a pungent oleogum resin known for its strong sulfurous aroma and valuable medicinal and nutritional properties. Traditionally used as both a spice and a folk remedy for centuries [14,15,16], recent studies have further demonstrated its antimicrobial effects [17,18,19,20,21,22]. Asafoetida has antimicrobial efficacy against pathogens such as *Salmonella typhi*, *E. coli*, *S. aureus*, *Bacillus subtilis*, *Aspergillus niger*, and *Candida albicans* [23,24,25]. Asafoetida is the dried latex extracted from the rhizome or taproot of several *Ferula* species, which are perennial herbs in the carrot family. This resin is primarily produced in regions such as Iran, Afghanistan, Central Asia, Northern India, and Northwest China.

In this study, we employed Asafoetida powder as a carbon source in a green one-step hydrothermal method to synthesize CQDs and copper-doped CQDs (Cu-CQDs). Then, their antimicrobial effects were evaluated both in solution and when attached to the surface of PVC plastic coupons as a proof of concept.

## 2. Materials and Methods

### 2.1. Materials

Anhydrous copper sulfate was obtained from CHIMIQUES ACS CHEMICALS Inc., Canada (99%). Absolute ethanol, n-hexane, heptane, and ethyl acetate were purchased from Sigma-Aldrich. Asafoetida was purchased from local supermarkets in Ahvaz, Khuzestan, Iran. Ultrapure water was used throughout the study. 3-(3-(trichlorosilyl)propoxy)propanoyl chloride (MEG-Cl) for CQD attachment to the surface of plastic coupons was pre-synthesized by the Thompson group [26]. LB and Agar were purchased from MedStore U of T (Toronto, ON, Canada). Experiments were carried out with *E. coli* GFP (ATCC 25922GFP), and *S. aureus* (KR3).

### 2.2. Instruments

Transmission electron microscopy (TEM) images were captured on a Hitachi HT7700 TEM/STEM at 80 kV after dispersing the CQDs on a carbon-coated copper grid. For atomic force microscopy (AFM), CQDs were dispersed in acetone, and a drop of the suspension was placed on a mica sheet, with imaging conducted in tapping mode using a Bruker Inspire (Multimode 8) equipped with a NanoWorld Arrow-NCPt tip (force constant = 42 N/m, resonance frequency = 285 kHz, tip radius < 25 nm). Particle size analysis (PSA) was performed using a Scatteroscope I, Qudix (Korea). Functional groups on the CQDs were identified using a Vortex 70 FTIR (Bruker, Germany), and UV spectra were recorded with a VWR 1600 PC UV–Vis spectrophotometer (China). Luminescence spectra and measurements were taken with a Thermo Scientific luminescence spectrometer (USA) and a PerkinElmer FL6500. XPS analysis was conducted with a Theta Probe Angle-Resolved X-ray Photoelectron Spectrometer System (Thermo Fisher Scientific Inc., Waltham, MA, USA) located at Surface Interface Ontario (University of Toronto, Toronto, ON, Canada). PVC coupons were imaged by fluorescence microscopy (Olympus BX63, Tokyo, Japan).

### 2.3. CQDs and Cu-CQDs Synthesis

CQDs and Cu-CQDs were prepared by hydrothermal method. About 100 mg of Asafoetida powder were dispersed in 40 mL ultrapure water in a Teflon linen of a stainless steel autoclave and the autoclave was tightened and heated in a muffle furnace at 200 °C for 4 h. As for Cu-CQDs synthesis, 30 mg anhydrous copper sulfate was completely dissolved in 40 mL of ultrapure water in the Teflon linen, and 100 mg Asafoetida powder was added and stirred for 5 min then Teflon linen was placed in a stainless steel body, sealed, and heated in an oven. After 4 h the reactor was cooled down, and the contents were centrifuged at 10,000 rpm to remove any large particles. A brown solution was obtained in both cases; this solution was dialysed into distilled water (3000 Dalton dialysis membrane); the dialysed samples were further purified by size exclusion chromatography using a 9 × 0.5 cm silica gel column.

### 2.4. Antibacterial Evaluation

Fifty microliters (50 µL) of a 1% bacterial culture of either *E. coli* or *S. aureus* from overnight culture in LB medium was added to each well of a 96-well plate. To this was added either 50 µL of 100 mg mL^−1^ ampicillin (Positive Control), 50 µL of deionized water (negative control), or 50 µL of QD solution which was serially diluted from full concentration to 1/64 concentration; each condition had 3 replicates. The well plate was then incubated at 37 °C with rotation at 120 rpm for 24 h; the solution from each well was diluted to 1 mL in deionized water and its absorbance at 600 nm was measured. This value was subtracted from each measurement to control for absorbance by the dots themselves. Three spots of each diluted solution (20 μL) was spotted onto an agar plate, and grown overnight at 37 °C. The appearance of colonies was then evaluated to determine the antibacterial properties of the CQDs.

### 2.5. Modification of Plastic

Plastic coupons (1 cm × 1 cm × 1.5 mm) were cut out from a PVC sheet. They were then rinsed with 1% solution of SDS in deionized water, followed by sonication for 5 min in the same solution. They were again rinsed with deionized water several times and finally rinsed with 95% ethanol solution. The coupons were then dried under a stream of nitrogen, followed by plasma cleaning in air (10 min per side). They were then stored in a humidity chamber (70% relative humidity) overnight.

The samples were transferred into pre-silanized test tubes, capped with rubber stoppers, and transferred into a glovebox with inert N_2_ atmosphere. A total of 1 mL of MEG-Cl solution in heptane (1:1000) was added to each sample, and the samples were stoppered and sealed with parafilm. They were removed from the glovebox and placed on a rotator at 120 rpm for 90 min. After this they were rinsed with heptane, and then sonicated for 5 min in heptane, and finally rinsed with 95% ethanol.

To each sample 1 mL of Cu-CQD solution (10.25 mg mL^−1^) was added in deionized water, to which 500 μL pyridine was added. The samples were rotated overnight, before the solution was removed and the samples were then rinsed with deionized water, followed by sonication in the deionized water for 5 min. The samples (plastic coupons) were then dried under a stream of air and stored in a clean vial until use.

### 2.6. Antibacterial Evaluation of Modified PVC

PVC samples with or without Cu-CQDs were exposed to bacteria and imaged according to previously published methods [27]. Each PVC coupon was placed in a well of a 24 well plate, and 1 mL of bacterial solution (1% *E. coli* from an overnight culture) in LB broth was added to the well. The plate was incubated at 37 °C for 24 h, after which each coupon was removed from its well and gently rinsed in saline solution (0.9% NaCl, 3 × 5 mL). 1% glutaraldehyde in saline solution was added to each plate (75 μL) and left for 30 min. The solution was removed from the plate, and 0.05% TWEEN-20 saline solution was added (75 μL) and left for 10 min. The solution was removed, and 0.1% Sytox Green saline solution was added (75 μL) and left for 30 min. The PVC samples were then rinsed with water (3 mL) and imaged using fluorescence microscopy.

## 3. Results and Discussion

### 3.1. Spectroscopic Evaluation of CQDs

**Figure 1:**
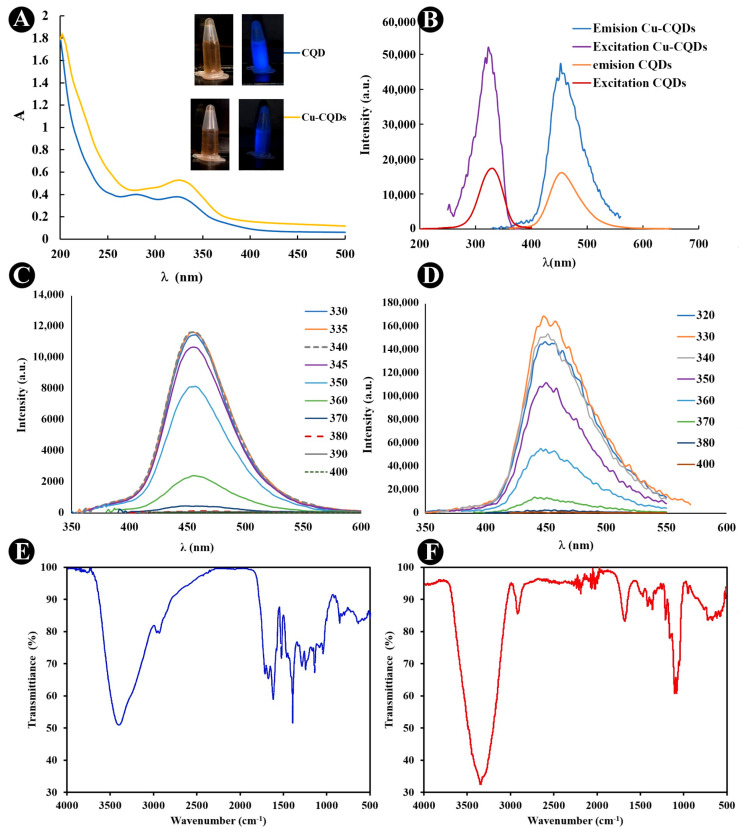
Spectroscopic evalutions of as-prepared CQDs and Cu-CQDs; (**A**) UV/Vis spectra; inset is the images of the dots under daylight and 365 nm UV; (**B**) excitation-emision profile; emission profile at different excitation wavelength for (**C**) CQDs and (**D**) Cu-CQDs; (**E**) FTIR spectra of CQDS; and (**F**) ATR-FTIR spectra of Cu-CQDs.

The UV/Vis, and fluorscence behavior of CQDs and Cu-CQDs are illustrated in Figure 1. It was observed that the water soluble carbon dots are brown in daylight conditions and produce blue light when irradiated by a 360 nm source. The blue emission of Cu-CQDs is more intense than that of the CQDs. The UV-Vis spectra (Figure 1A) of both particles are sligthly different, which reflcts an indication of copper doping of the carbon quantum dots. CQDs have two absorption maxima at 280 nm and 335 nm but Cu-CQDs have an absorption maxima at 335 nm. The absorption band at around 340 nm in carbon dots is often associated with *n→π* transitions, typically attributed to the presence of surface functional groups or defects containing oxygen- or nitrogen-containing groups, such as C=O (carbonyl) or N-H groups. These groups can form energy states within the carbon dot structure that absorb light in the UV range, around 340 nm [28,29]. One study reported how the presence of specific functional groups, like C=O and C–OH, alters the optical and photoluminescent properties, leading to characteristic UV-visible absorption bands (typically in the 260–340 nm range) due to n→π* and π→π* transitions [13]. Additionally, this absorption band can indicate both surface and defect sites that play a role in the electronic structure of CQDs, often contributing to their photoluminescent properties and possibly influencing their antibacterial activity. By modifying the functional groups or surface passivation of CQDs, this absorption peak can often be tuned, affecting the CQDs’ emission properties and interactions with biological targets [30]. Direct optical band gap of CQDs and Cu-CQDs determined by Tauc plots using the corresponding UV/Vis spectra [31,32] were in the range of 3.39–4.35 eV, and 3.46–4.65 eV, respectively. No significant changes in the band gaps were observed on Cu doping indicating that both particles are of approximately the same size.

Both a quantum effect and surface functional groups determine the photoluminescence properties of the quantum dots [11,29,30]. The fluorescence emission of CQDs and Cu-CQDs (Figure 1B–D) shows an excitation wavelength independent of the emission profile indicating that uniform surface states are available in as-prepared dots. Maximum emission intensity is observed at 450 nm and 455 nm, when excited at 335 nm and 330 nm for CQDs and Cu-CQDs, respectively. The emission intensity is higher in Cu-CQDs (Figure 1B). The fluorescence quantum yield for CQDs and Cu-CQDs were 37% and 73.4%, respectively (for the details of quantum yield determination see the supplementary material). When CQDs are doped with copper, highly luminescent carbon dots are produced. A higher quantum yield may indicate a more rigid structure of CQDs due to copper interaction with the functional groups [33]. Copper doping also introduces Cu-related electronic states and modifies surface chemistry of CQDs. These new states promote radiative recombination while passivating nonradiative traps; together they increase the radiative fraction of excited carriers and thus the photoluminescence quantum yield [34].

X-ray photoelectron spectroscopy (XPS) provides valuable insights into the luminescence properties of CQDs by analyzing their surface chemistry and electronic structure. XPS detects functional groups or defects on the CQD surface. These surface features can serve as sites for electron or hole trapping, impacting the CQDs’ emission properties. For instance, oxygen-containing functional groups often enhance photoluminescence by creating trap states that contribute to radiative recombination. XPS also reveals shifts in binding energy, indicating changes in electronic structure. Shifts in binding energies can suggest stronger quantum confinement or altered electronic interactions, which can lead to increased or shifted luminescence wavelengths. For example, if copper doping causes a shift in binding energies, it could be linked to enhanced rigidity in the CQD structure, contributing to higher luminescence stability.

**Figure 2:**
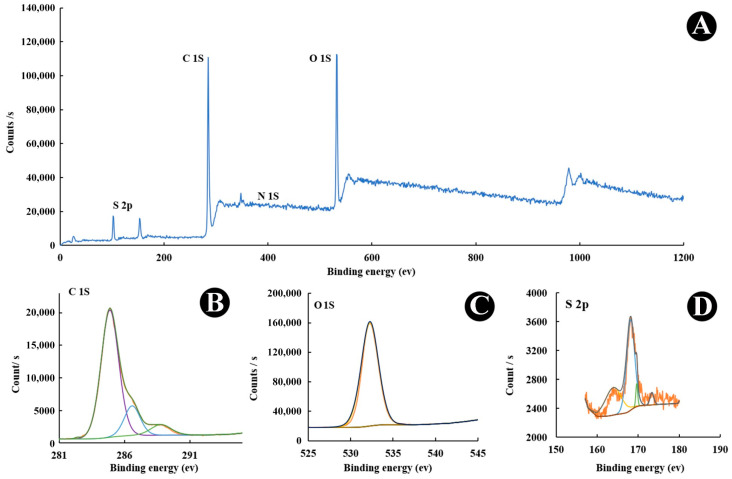
(**A**)X-ray photoelectron spectroscopy (XPS) survey, (**B**) C1s, (**C**) O1s, and (**D**) S2p of CQDs.

**Figure 3:**
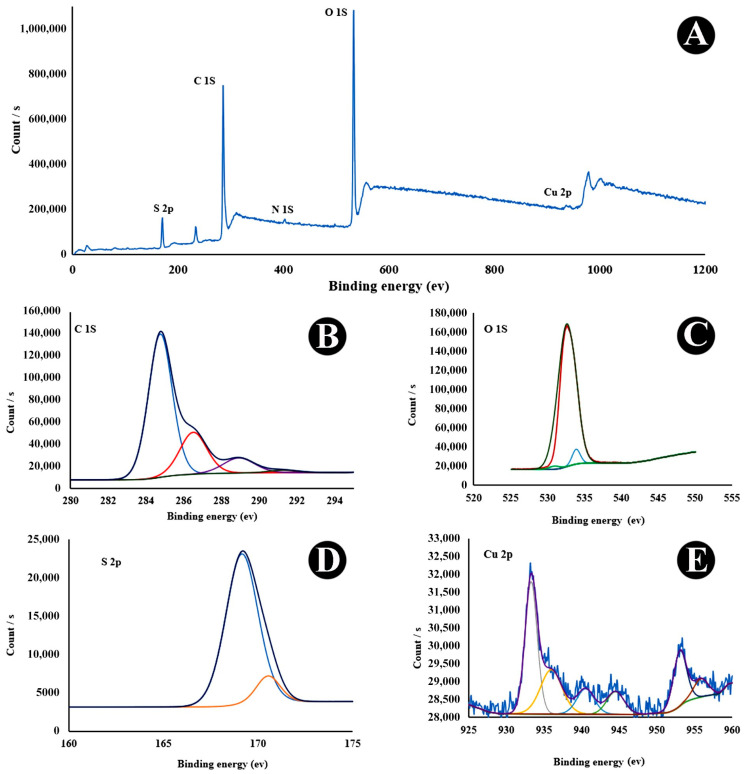
(**A**) X-ray photoelectron spectroscopy (XPS) survey, (**B**) C1s, (**C**) O1s, (**D**) S2p, and (**E**) Cu2p of Cu-CQDs.

Figure 2 and Figure 3 present the XPS spectra for as-prepared CQDs and Cu-CQDs, respectively. Comparison of the two surveys reveales that both Cu and N peaks are observed in Cu-CQDs. O 1s in CQDs (Figure 2) and Cu-CQDs. Figure 3 has a peak in the range of 526–540 eV that has a maximum arund 532.38 eV which originates from an O 1s signal. This is likely associated with interfacial oxygen-containing functionalities, primarily C–O and C=O groups, present on the CQDs surface. The C 1s spectrum shows three main binding energies at 284.8, 286.5, and 288.5 eV, which are assigned to the graphitic core/sp^2^–sp^3^ carbon (C–C/C–H), C–O, and carbonyl/carboxyl (C=O/O–C=O) groups, respectively. The S 2p peak at 163.8 eV is attributed to the C–S–C bonding in the CQDs. After Cu incorporation, the S 2p signal shifts to a higher binding energy, indicating the formation of Cu^2+^–S bonds (Figure 3). This is attributed to electron withdrawal by Cu^2+^, which reduces the electron density around sulfur atoms [33,35]. Another noticeable change in the XPS spectra is the increase in the N 1s peak around 400 eV, which suggests that Cu also coordinates with nitrogen sites. The nitrogen and sulfur detected in the CQDs originate from natural plant-derived precursors, such as proteins or amino acids, which act as inherent sources of heteroatoms during carbonization.

FTIR spectroscopy also confirms the functional groups (Figure 1E,F)). Peaks at 1611, 1515, and 837 confirm the aromatic graphene core, and Sp^2^-hybridized carbon structure, respectively. OH (3409 cm^−1^) and COOH (1707 cm^−1^) peaks are also observed in FTIR spectra of both particles with a few nanometer shifts in Cu-CQDs due to copper compexation. The Zeta potential of CQDs and Cu-CQDs were 5.95 mV and −27.1 mV, consequently. Accordongly, Cu-CQDs possess higher stability of suspension which results in an increased interaction with bacteria.

### 3.2. Microscopic Evaluations of CQDs

**Figure 4:**
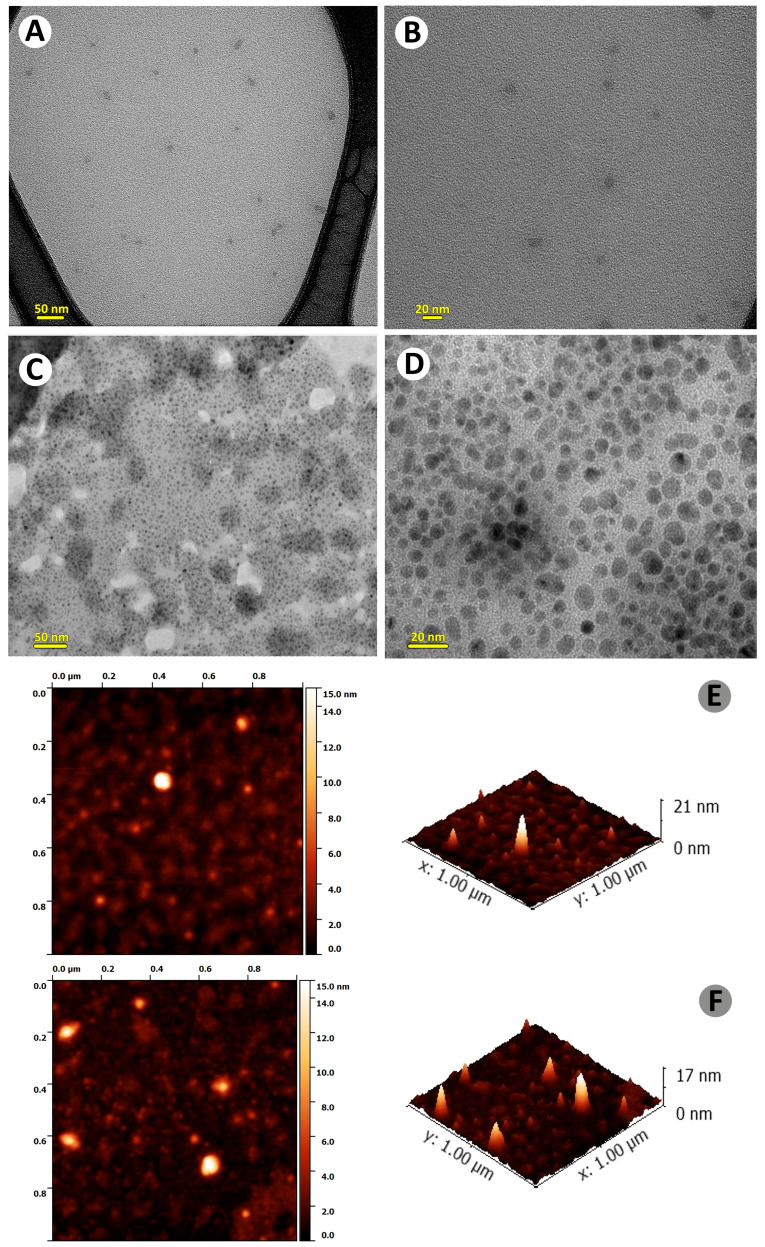
TEM images of (**A**,**B**) CQDS and (**C**,**D**) Cu-CQDS; atomic force microscopic images (AFM) of (**E**) CQDs and (**F**) Cu-CQDs.

TEM and AFM are commonly used techniques to analyze morphology, size, and surface characteristics of nanoparticles. TEM and AFM images of CQDs and Cu-CQDs (Figure 4) confirms the formation of quantum dots. TEM provides high-resolution images of CQDs, typically showing spherical or quasi-spherical nanoparticles with sizes in the range of 1.6–4.3 nm (Figure 4A) where most particles are 1.6 nm (about 50%). The presence of copper on CQDs (Figure 4A) provides better contrast, allowing clearer TEM images. In addition, the size of Cu-CQDs has increased to 3.9–7.3 nm. The majority of particles have sizes below 5.6, with most frequent particle sizes ranging from 3.9 to 5.6 nm.

AFM reveals surface topography and height. Bright spots in AFM indicates the CQDs (Figure 4B) and height profile shows their thickness. In AFM 3D surface image, height distribution is observed. A uniform height distribution suggests monodispersed particles, while variation indicates aggregation or non-uniform functionalization. CQDs with a thickness of 1–3 nm suggests a single or few-layered graphene-like structure. If the thickness is higher (>3 nm), it indicates stacked or functionalized CQDs [36]. Figure 4B shows some degree of aggregation of both CQDs. In addition, the higher thickness of some particles coated on the mica sheet for Cu-CQDs could be an indication of functionalization or more aggregation in Cu-CQDs. The EM image of the Cu-CQDs also shows some degree of aggregation [29].

### 3.3. Antibacterial Activities of As-Prepared CQDs

**Figure 5:**
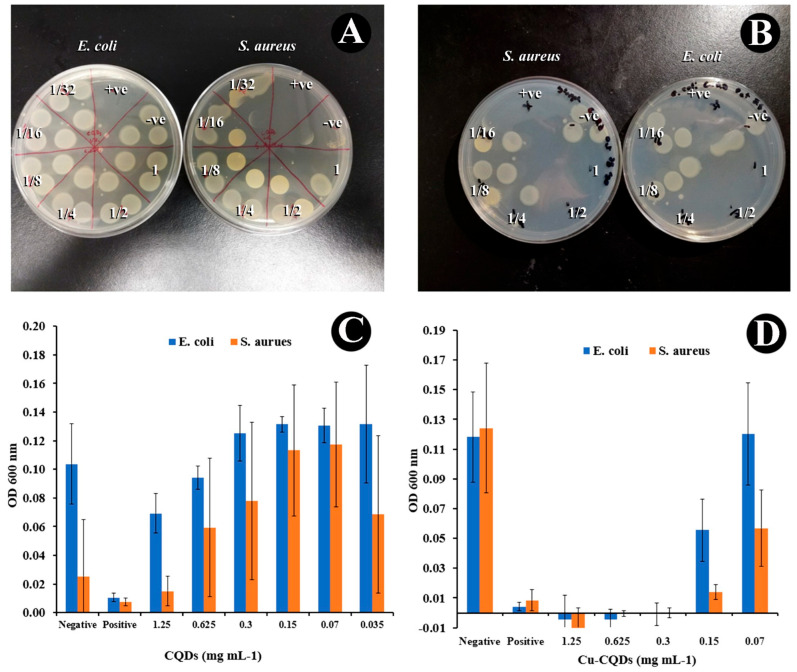
Effect of (**A**) CQDs and (**B**) Cu-CQDs on bacterial growth inhibition on agar plate for both *E. coli* and *S. aureus* Optical density (OD 600 nm) measurements of [*E. coli*] and [*S. aureus*] cultures treated with (**C**) CQDs and (**D**) Cu-CQDs at varying concentrations (1.25–0.035 mg mL^−1^). Negative (water) and positive (penicillin) controls are included for baseline comparison.

As a proof of concept, the antibacterial activity of CQDs and Cu-CQDs against *E. coli* as Gram-negative bacteria and *S. aureus* as representative of a Gram-positive bacteria was evaluated by both agar plate and solution methods. As indicated in Figure 5 both particles have higher antibacterial activity against *S. aureus*. The antibacterial activity of Cu-CQDs is much higher than it is for CQDs (Figure 5B). Plating of bacterial samples on agar resulted in either no bacteria being visible, or oversaturating of the spots with bacteria. Thus the CFU for each dilution was either 0, or uncountable. For CQDs without copper, *E. coli* showed complete oversaturation of the spots at each tested dilution, while for *S. aureus* there was only one saturated spot out of 3 visible at 1.25 mg mL^−1^ CQD concentration, compared to 3 fully saturated spots for all other dilutions. For Cu-CQDs, no cell growth was visible on the plates for concentrations of CuCQD-s in the range of 0.3–1.25 mg mL^−1^ for either bacteria. At a concentration of 0.15 mg mL^−1^ there was a single saturated spot visible for *S. aureus*, and a single colony visible for *E. coli*. At lower concentrations of Cu-CQDs each sample showed 3 over-saturated spots, suggesting little to no antibacterial properties at these lower concentrations.

The graphs in Figure 5 represent the optical density (OD 600 nm) for *E. coli* (blue bars) and *S. aureus* (orange bars) under different treatments with CQDs and Cu-CQDs, at varying concentrations. The positive and negative controls are penicillin and distilled water, respectively. No complete inhibition is observed for CQDs, as OD values remain relatively high. CQD 1.25 mg mL^−1^ shows the lowest bacterial growth, but inhibition is incomplete for both *E. coli* and *S. aureus*. Cu-CQD up to 0.3 mg mL^−1^ has zero OD values, indicating nearly complete bacterial inhibition for both Gram-positive and Gram-negative bacteria. Lower concentrations (e.g., 0.15 mg mL^−1^ and less) show increased bacterial growth, while 0.07 mg mL^−1^ Cu-CQDs still show less than 50 percent growth inhibition for *S. aureus*. The minimum inhibitory concentration (MIC) appears to be 0.3 mg mL^−1^ (±0.01) for Cu-CQDs, as this is the lowest concentration showing complete bacterial inhibition. CQDs do not achieve full inhibition at any tested concentration but show a reduction in bacterial growth at 1.25 mg mL^−1^.

*E. coli* is a Gram-negative bacterium with a thin peptidoglycan layer (3–6 nm) and an outer membrane containing lipopolysaccharides (LPS), which makes it more resistant to antibiotics. In contrast, *S. aureus* does not have an outer membrane but has a thick peptidoglycan layer (20–30 nm) [37,38]. The Zeta potential of CQDs is 5.95 mV suggesting electrostatic attraction of CQDs to bacterial cell walls with *S. aureus*’s thick peptidoglycane layer providing more binding sites for CQDs compared to *E. coli*’s thin peptidoglycan and outer LPS membrane. Therefore, a lower activity of CQDs against *E. coli* compared to *S. aureus* is observed [3,7]. Cu-CQDs with their higher zeta potential of −27.1 mV show higher colloidal stability and better contact with the bacteria.

Copper exhibits strong antibacterial activity against both Gram-positive (*S. aureus*) and Gram-negative (*E. coli*) bacteria through multiple mechanisms that disrupt bacterial survival. Despite the electrostatic repulsion due to the high negative zeta potential of Cu-CQDs (−27.1 mV), *S. aureus* interacts better because the thick peptidoglycan layer provides more binding sites for Cu-CQDs compared to *E. coli.* In addition, the presence of negatively charged Teichoic acid in the *S. aureus* cell wall attracts Cu^2+^ facilitating localized ion accumulation and membrane damage [6]. Accordingly, Cu^2+^ ion release and physical disruption may be considered as the main antibacterial mechanism. Nanoscale Cu-CQDs penetrate biofilms and bacterial cell wall via partial LPS membrane disruption in *E. coli* and direct peptidoglycan layer penetration for *S. aureus* due to small pore size in the cell wall. The presence of surface Cu^2+^ on CQDs can undergo redox cycling (Cu^2+^ ⇄ Cu^+^) in the bacterial microenvironment, especially in the presence of intracellular reductants (thiols). Cu-CQDs derived from Asafoetida have a lower MIC (0.3 mg mL^−1^, see Table S1 for more details) compared to plant extract in different solvents, which have MIC values of 1 and 2 mg mL^−1^ [24].

*E. coli* removal from catheter surfaces is crucial for several reasons. First its entire role in catheter-associated urinary tract infections (CAUTIs) and other healthcare-associated infections are well-known [39,40,41]. *E. coli* is one of the primary pathogens responsible for CAUTIs. It can form biofilms on catheter surfaces, creating a protective environment that makes the bacteria more resistant to antibiotics and host immune responses. Biofilm-associated infections are difficult to treat and often lead to persistent infections. Biofilm-forming *E. coli* can also develop antibiotic resistance, making infections harder to treat. Catheter-related infections can lead to severe complications, including bloodstream infections and sepsis. Eliminating *E. coli* from catheter surfaces reduces the risk of systemic infections and associated morbidity and mortality. Preventing bacterial adhesion and biofilm formation can also help reduce the need for antibiotics and slow the emergence of resistant strains. Bacterial colonization can lead to catheter blockages and malfunction, necessitating frequent replacements. Preventing *E. coli* adhesion helps maintain catheter function and reduces the need for repeated interventions [40,41,42].

**Figure 6:**
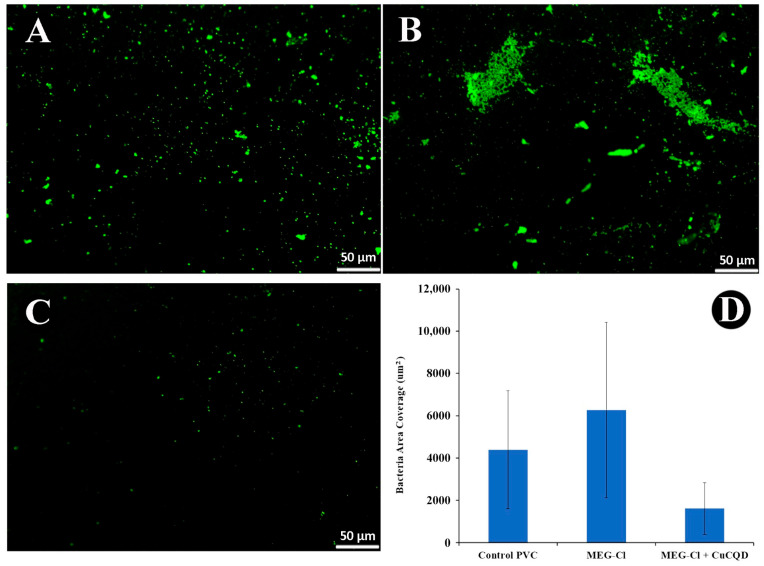
*E. Coli* growth pattern on (**A**) the bare PVC coupon, (**B**) the PVC modified with MEG-Cl, and (**C**) MEG-Cl-Cu-CQDs. (**D**) bacterial counts against each coated surface.

Given these factors, Cu-CQDs were attached to the surface of polyvinyl chloride to evaluate *E. coli* growth in comparison with the uncoated PVCs samples. The PVC coupons were observed under a fluorescence microscope and the number of bacteria after 24 h of incubation at 37 °C was counted. As illustrated in Figure 6, *E. coli* growth was significantly reduced. This means that these dots can potentially act as an antimicrobial agent without light activation. Such a result could lead to potential use as a coating for catheters and other medical devices.

Recently, thermoplastic elastomer doped with CQDs has been introduced as antibacterial material suitable for catheters after one hour of exposure to blue light [43]. Additionally, Cu-CQDs demonstrated significant antibacterial activity (Figure 6) against *E. coli* even in the absence of light, making them highly desirable and easily applicable in catheter materials.

## 4. Conclusions

This study demonstrates a green and efficient hydrothermal synthesis of antibacterial carbon quantum dots (CQDs) from Asafoetida, yielding CQDs with excellent quantum yield while preserving bioactive surface groups that enhance antibacterial performance. Copper doping further improved activity by promoting stronger interaction with bacterial biofilms, resulting in potent inhibition of *S. aureus*. In addition, Cu-CQDs immobilized on PVC surfaces effectively prevented bacterial attachment and biofilm formation, highlighting their potential as eco-friendly antimicrobial coatings for catheters and other medical devices. These findings introduce a promising strategy for long-term infection control and represent the first report of CQD immobilization on plastic substrates for antibacterial and antifouling biomedical applications without light activation.

## Data Availability

The data presented in this study are available on request from the corresponding author.

## Supplementary Materials

The following supporting information can be downloaded at: https://www.mdpi.com/article/10.3390/nano15231804/s1, Figure S1: Tuct plot for determination of band gap of CQDs and Cu-CQDs. Excell data points are available on request; Table S1: Optical density at 600 nm for the solution of bacteria and carbon dots.

## Abbreviations

The following abbreviations are used in this manuscript:

AFM	Atomic Force Microscope
TEM	Transmission Electron Microscope
CQDs	Carbon quantum dots
Cu-CQDs	Copper-doped carbon quantum dots
CAUTIs	catheter-associated urinary tract infections
PVC	Poly vinyl chloride
MEG-Cl	3-(3-(trichlorosilyl)propoxy)propanoyl chloride
ROS	Reactive oxygen species
LPS	Lipopolysaccharides
MIC	minimum inhibitory concentration
XPS	X-ray photoelectron spectroscopy
GFP	Green fluorescence protein
