# Supplementary material for: Green Synthesis of Highly Luminescent Carbon Quantum Dots from Asafoetida and Their Antibacterial Properties
Source: Nanomaterials (Basel). 2025 Nov 29;15(23):1804. doi: 10.3390/nano15231804 (PMC12693027; doi:10.3390/nano15231804)
Supplement: Supplementary file 1 [file nanomaterials-15-01804-s001.zip › nanomaterials-3986192-supplementary.pdf]

Supplementary materials

Green Synthesis of Highly Luminescent Carbon Quantum Dots from Asafoetida and Their Antibacterial Properties

Zahra Ramezani [1,*], Armita Khayat [1], Brian De La Franier [2], Abdolghani Ameri [3] and Michael Thompson [2,*]

[1] Nanotechnology Research Center, Medical Basic Sciences Research Institute, Ahvaz Jundishapur Univesity of Medical Sciences, Ahvaz 61357-15794, Iran
[2] Department of Chemistry, University of Toronto, 80 St George S., Toronto, ON M5S 3H6, Canada
[3] Department of Food and Drug Control, Ahvaz Jundishapur University of Medical Sciences, Ahvaz 61357-15794, Iran
* Correspondence: zramezani@ajums.ac.ir (Z.R.); m.thompson@utoronto.ca (M.T.)

Abstract

Highly Luminescent carbon quantum dots (CQDs) and copper-doped CQDs (Cu-CQDs) were synthesized from Asafoetida powder using a one-pot hydrothermal method. The structural, morphological, and optical properties of the synthesized CQDs were characterized via microscopic and spectroscopic techniques. Photoluminescence studies revealed that CQDs exhibited maximum emission at 450 nm under 335 nm excitation with a quantum yield of 37%, while Cu-CQDs showed a red-shifted emission at 455 nm under 330 nm excitation and a significantly enhanced quantum yield of 73.4%. As proof of concept for potential biomedical and surface-coating applications, the antimicrobial activity of both CQDs was evaluated against *Escherichia coli* (*E. coli*) and *Staphylococcus aureus (S. Aureus)*. Cu-CQDs exhibited superior antibacterial efficacy, with a minimum inhibitory concentration of 0.3 mg/mL. Furthermore, Cu-CQDs were immobilized on polyvinyl chloride (PVC) surface, and fluorescence microscopy confirmed their antibacterial effectiveness, demonstrating their potential for functionalized antimicrobial coatings.

Methods:

Quantum yield determination

An absolute method using quinone sulphate as standard was used for QY measurements. Quinone sulfate in 0.1M sulfuric acid has quantum yield 0.54 (54%) excited at 335 nm. It was used as standard for quantum yield determination by Equation (S1)

$$Qu = Qs\frac{Fu}{Fs}\frac{As}{Au}\frac{\eta u}{\eta s}$$ Equation (S1)

Where, Φs and Φu stand for quantum yield of standard and as prepared CQDs and Cu-CQDs, consequently. F, A, η stand for fluorescence intensity, absorbance at the excitation wavelength, and refractive index, respectively. Solutions of quinone sulfate and CQDs having absorbance below 0.05 were excited at 335 nm, and their emission at 450 nm was recorded. Then, the quantum yield was determined by replacing the corresponding values in Equation S1. Refractive indexes were determined for CQDs, Cu-CQDs, and quinone sulfate.

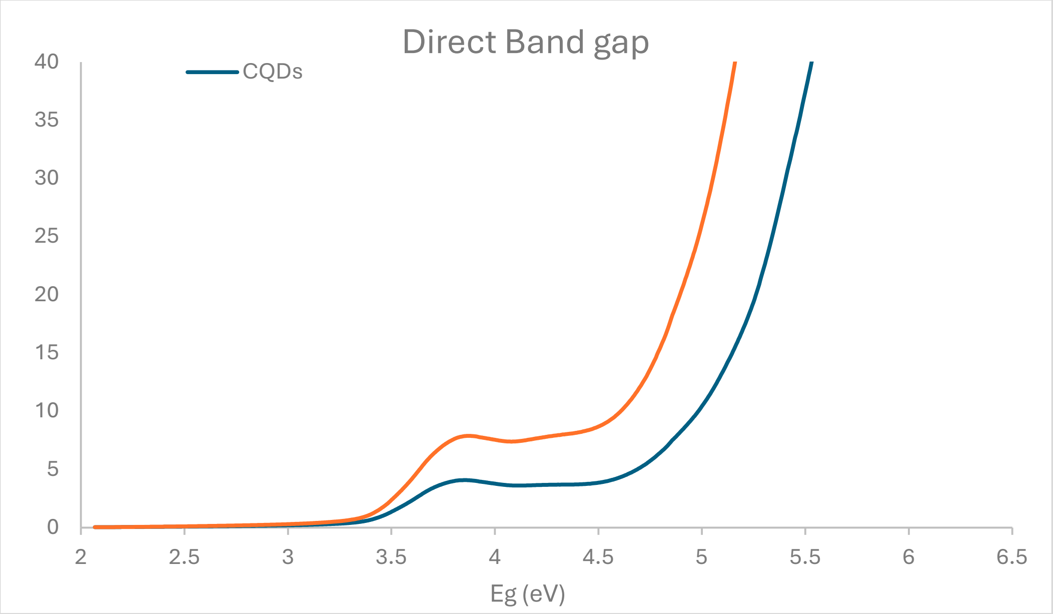

Figure S1 Tuct plot for determination of band gap of CQDs and Cu-CQDs. Excell data points are available on request.

Table S1 shows optical density measurement at 600 nm for solutions of CQDs and Cu-CQDs with *E. coli* and *S. Aureus*.

Table S1 Optical density at 600 nm for the solution of bacteria and carbon dots. All data are mean of three measurements.

	E. Coli		S. Aureus	
mg mL^{-1}	CQDs	Cu-CQDs	CQDs	Cu-CQDs
1.25	0.07±0.01	0.00±0.02	0.02±0.01	-0.01±0.02
0.625	0.09±0.01	0.00±0.00	0.06±0.05	-0.00±0.00
0.3	0.13±0.02	0.00±0.00	0.08±0.04	0.00±0.00
0.15	0.13±0.01	0.06±0.02	0.11±0.05	0.02±0.00
0.07	0.13±0.04	0.12±0.03	0.12±0.05	0.06±0.03
